# Hour-Ahead Photovoltaic Power Prediction Combining BiLSTM and Bayesian Optimization Algorithm, with Bootstrap Resampling for Interval Predictions

**DOI:** 10.3390/s24030882

**Published:** 2024-01-29

**Authors:** Reinier Herrera-Casanova, Arturo Conde, Carlos Santos-Pérez

**Affiliations:** 1 Faculty of Mechanical and Electrical Engineering, Autonomous University of Nuevo León, San Nicolás de los Garza 66455, Mexico; herreracasanovareinier@gmail.com (R.H.-C.); con_de@yahoo.com (A.C.); 2 Department of Signal Theory and Communications, University of Alcalá, 28805 Alcalá de Henares, Madrid, Spain

**Keywords:** photovoltaic power forecasting, bidirectional long short-term memory (BiLSTM), predictions intervals, Bayesian optimization

## Abstract

Photovoltaic (PV) power prediction plays a critical role amid the accelerating adoption of renewable energy sources. This paper introduces a bidirectional long short-term memory (BiLSTM) deep learning (DL) model designed for forecasting photovoltaic power one hour ahead. The dataset under examination originates from a small PV installation located at the Polytechnic School of the University of Alcala. To improve the quality of historical data and optimize model performance, a robust data preprocessing algorithm is implemented. The BiLSTM model is synergistically combined with a Bayesian optimization algorithm (BOA) to fine-tune its primary hyperparameters, thereby enhancing its predictive efficacy. The performance of the proposed model is evaluated across diverse meteorological and seasonal conditions. In deterministic forecasting, the findings indicate its superiority over alternative models employed in this research domain, specifically a multilayer perceptron (MLP) neural network model and a random forest (RF) ensemble model. Compared with the MLP and RF reference models, the proposed model achieves reductions in the normalized mean absolute error (nMAE) of 75.03% and 77.01%, respectively, demonstrating its effectiveness in this type of prediction. Moreover, interval prediction utilizing the bootstrap resampling method is conducted, with the acquired prediction intervals carefully adjusted to meet the desired confidence levels, thereby enhancing the robustness and flexibility of the predictions.

## 1. Introduction

### 1.1. Background and Motivation

In recent years, solar photovoltaic (PV) has emerged as a widely used energy source worldwide. According to the International Renewable Energy Agency (IRENA), the global installed capacity of solar PV is expected to increase significantly in the coming years, reaching 2840 GW in 2030 and 8519 GW in 2050 [1]. Consequently, the significance and penetration of this type of energy in power grids are expected to elevate.

The dynamics of solar energy are intricately linked to various climatic factors, including solar irradiance, cloudiness, temperature, relative humidity, and wind. Additionally, PV energy production is influenced by the geographical location of the solar installation, seasonal variations throughout the year, and the alternation between days and nights. The varying magnitudes of these factors contribute to the fluctuating and intermittent nature of PV energy, presenting challenges for its seamless integration into electrical grids [2,3].

Several studies have addressed the main challenges associated with the integration of PV power into electrical systems. In [4,5], it is emphasized that frequency stability is considered a significant technical challenge restricting the integration of solar energy at the transmission level. References [5,6] highlight that the intermittency of PV power can also lead to voltage issues (under/overvoltage, voltage fluctuation and voltage unbalance). High ramp rates, mainly associated with the passage of clouds, result in sudden changes in power generation over relatively short periods of time [5]. Moreover, challenges may arise related to the adjustment of protection devices, reverse power flows, planning and economic dispatch problems, increased reserve levels, and flexibility requirements, among others [6].

Power system operators underscore the imperative to minimize the level of uncertainty associated with this energy source for facilitating its grid integration. In this context, accurate forecasting of PV energy production at different temporal and spatial scales proves highly valuable in addressing the aforementioned challenges [7,8]. Precision in PV power forecasting directly impacts practical aspects related to grid planning, management of system reserve levels, and economic dispatch of generators. Operationally, it ensures voltage and frequency stability of the grid, regulation and control of ramp events, among other crucial aspects. As the number of PV systems installed worldwide increases, accurate forecasting of their performance will become increasingly important for efficient power system management. Despite the great importance of PV power forecasts, obtaining them remains a challenging and highly complex task. It involves predicting the behavior of a highly variable power source, responding to highly random and uncertain weather conditions [9].

### 1.2. Literature Review

**Figure 1:**
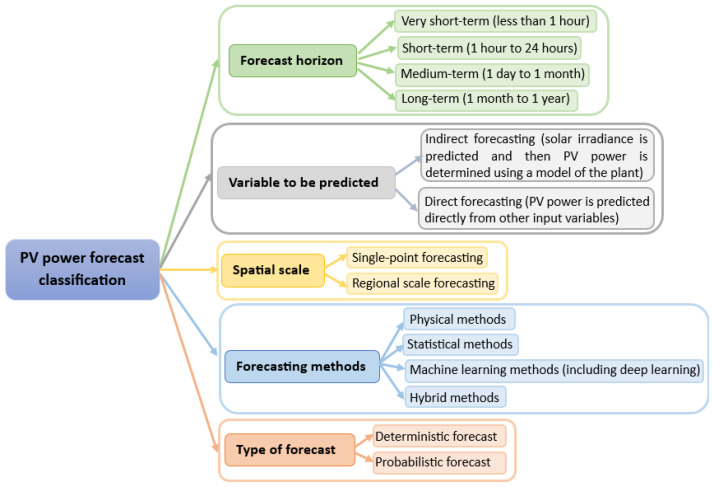
General classification of PV energy forecasts.

PV energy forecasts exhibit various classifications depending on crucial factors like prediction horizon, the variable under prediction, spatial scale, forecast methodology, and forecast type. Figure 1 offers a comprehensive summary of the categorization of PV energy forecasts.

Over the past decade, the field of PV energy prediction research has experienced significant growth, marked by a particular interest in the application of various machine learning (ML) and deep learning (DL) models. In [10], deterministic PV power prediction is performed using a long short-term memory (LSTM) model, demonstrating the superiority of the LSTM model compared with other ML models in this task. A comparison of some statistical models and different types of artificial neural networks (ANNs) is presented in [11], showing that ANN-based models are more accurate in predicting PV one hour in advance. In [12], an iterative filtering (IF) method and an extreme learning machine (ELM) are combined, demonstrating that the use of multiple lags of the PV power time series improves result quality. Reference [13] also achieves good results in very short-term solar energy forecasting by combining a multilayer perceptron (MLP) neural network model and the RRelieff method to select the most relevant input features.

In [14], an MLP model and numerical weather prediction (NWP) model are used to predict short-term PV energy, emphasizing the importance of efficient preprocessing of the available data. Reference [15] compares four LSTM model architectures (vanilla, stacked, encoder-decoder, and bidirectional long short-term memory (BiLSTM)) in an hour-ahead PV prediction, demonstrating the superiority of the BiLSTM architecture in terms of accuracy. In [16], a nonlinear autoregressive neural network with exogenous inputs (NARX) model is optimized using a genetic algorithm (GA) for very short-term PV energy forecasting, proving the effectiveness of the proposed strategy. Reference [17] presents a hybrid NARX-LSTM model where relevant hyperparameters are adjusted by a tabu search algorithm (TSA), achieving excellent results for deterministic and interval forecasts in very short-term and short-term horizons, although with a high computational cost.

In [18], PV energy is predicted for a very short-term horizon using an LSTM model. The proposed prediction strategy uses locally obtained sky images and historical PV energy records as inputs. The comparison with other benchmark models demonstrates the effectiveness of the proposed approach. However, its applicability may be limited due to the dependence on sky images obtained with specialized equipment. Reference [19] combines a support vector machine (SVM) model with an improved ant colony optimization (IACO) algorithm to predict PV power with a horizon of 30 min to 6 h ahead, showcasing improved predictions through hyperparameter optimization. In [20], a very short-term prediction of solar power is performed using a convolutional neural network (CNN) model consisting of four kernels. The authors show how the quality of the predictions degrades with increasing forecast horizon. In [21], a GA and a BiLSTM model are combined for very short-term PV energy forecasting, demonstrating the strength of this type of model in forecasting complex and nonlinear time series. In [22], a gated recurrent unit (GRU) model is used to predict solar irradiance, with tests showing that the inclusion of cloud cover data and other exogenous variables enhances forecast quality.

A combination of BiLSTM and CNN models is presented in [23], each specialized in extracting specific features from input data, significantly improving prediction accuracy, although with increased complexity. In [24], a GRU model is combined with a variational mode decomposition (VMD) method and an improved sparrow search algorithm (ISSA) for hyperparameter optimization, ensuring good performance under different operating conditions. In [25], a DL model redesigns the input gates of a GRU model using convolutional layers to improve the learning process. In turn, reference [26] proposes deterministic prediction models based on ensemble algorithms, specifically random forest (RF) and gradient boosting machine (GBM), recommending them as suitable alternatives for online prediction applications.

### 1.3. Contributions

From the literature analysis, it is appreciated that many researchers employ ML or DL models for PV solar energy forecasting. The use of hybrid models, combining various simple models, is acknowledged to positively impact prediction quality. However, some studies propose highly complex models that require extended training times. In many cases, extensive datasets are used, involving variables that are challenging to obtain, and model performance is not validated on smaller datasets, which is typical for PV installations operating over a relatively short period. Consequently, their applicability in other operational scenarios may be limited. Additionally, it is observed that, in some instances, data preprocessing has not received the necessary attention, despite its significant role in improving predictive model performance. While literature demonstrates that hyperparameter optimization can enhance prediction model accuracy, some studies perform this adjustment using a trial-and-error approach, consuming considerable time without guaranteeing the most suitable fit. Another notable aspect is that most studies focus on deterministic predictions, with few incorporating probabilistic prediction approaches to manage uncertainties in solar energy predictions.

The forecasting strategy proposed in this research relies on a BiLSTM type DL model, utilized for a direct prediction of PV power for the next hour based on historical data from a PV installation. The primary contributions of this research are as follows:

(i) Extensive and thorough preprocessing of the available dataset compared with other literature works, with results validating the robustness and effectiveness of the proposed algorithm.
(ii) Introduction of a hybrid prediction model (BiLSTM-Bayesian optimization algorithm (BOA)) using optimized combinations of hyperparameters to enhance the predictive capability; tests on reduced datasets corresponding to different seasons demonstrate the quality of the obtained results.
(iii) Comparative study between the BiLSTM model and other widely used prediction models in the literature (MLP and RF), showing that under similar conditions, the BiLSTM model exhibits superior performance in predicting PV power one hour in advance.
(iv) Application of a forecasting strategy combining deterministic predictions from the proposed model with interval predictions obtained through the bootstrap resampling method, thereby increasing prediction flexibility and appropriately characterizing uncertainties.

### 1.4. Paper Structure

The rest of the paper is organized as follows. Section 2 introduces the proposed framework for hour-ahead PV power forecasting and describes the fundamental aspects that comprise it. Section 3 and Section 4 analyze the aspects related to deterministic and interval predictions, respectively, and present the main results obtained in each case. The conclusions are finally highlighted in Section 5.

## 2. Hour-Ahead PV Power Forecasting Framework

**Figure 2:**
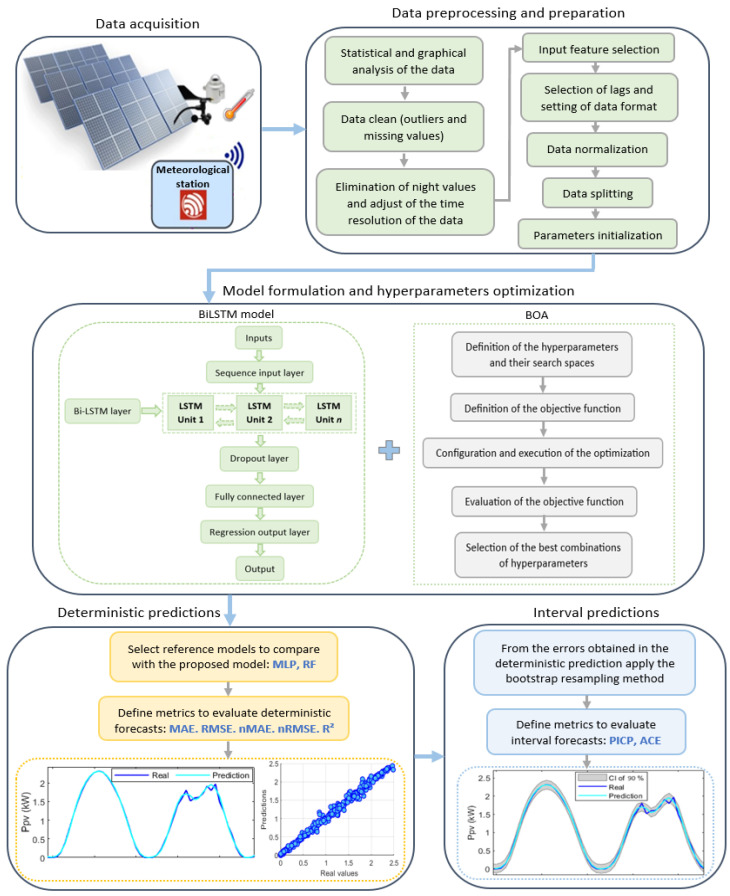
Proposed framework for PV power forecasting.

The proposed hour-ahead forecasting strategy is described in Figure 2. This strategy is composed of five fundamental blocks or stages: (i) data acquisition; (ii) data preprocessing and preparation; (iii) model formulation and hyperparameter optimization; (iv) deterministic predictions; and (v) interval predictions. The following sections describe in detail each of the above stages and their main characteristics.

### 2.1. Data Acquisition

The initial phase of the proposed forecasting strategy addresses the intricacies surrounding the acquisition of historical data essential for the development of the prediction model. In this instance, data are sourced from a modest 2.97 kW PV installation situated at the Polytechnic School of the University of Alcala in Spain [27]. Comprising 9 polycrystalline silicon solar modules of 330 W each, this installation features an on-site weather station equipped with various measuring devices. These devices record crucial variables such as solar irradiance, wind speed and direction, relative humidity, ambient temperature, and panel temperature. Additionally, measurements of the generated PV power at the facility are available [27,28]. It is noteworthy that the data’s temporal resolution is set at 15-min intervals, covering only the time span from 1 January 2021, to 31 December 2022. Reference [27] offers a comprehensive explanation of the PV installation under study and the measurement devices employed to record each of the mentioned variables.

### 2.2. Data Preprocessing and Preparation

The second phase of the forecasting strategy focuses on data preprocessing and preparation, addressing pertinent aspects related to the data. This stage covers the following aspects: statistical and graphical analysis of the data; data cleaning (including correction of outliers and missing values); elimination of night values and adjustment of the temporal resolution of the data; selection of input features to the prediction model; selection of lags in the input time series and adjustment of the data format; data normalization; and finally data splitting. Proper data preprocessing is crucial to ensure optimal performance of prediction models, facilitating improved convergence, reduced computational costs, and enhanced prediction accuracy.

#### 2.2.1. Statistical and Graphical Data Analysis

Conducting statistical and graphical analysis of the data is of great importance to gain a general understanding of the trends and characteristics of the dataset under study. Table 1 provides a comprehensive overview of the primary statistical indicators related to each variable under analysis.

In general, it is evident that the logical boundaries of each variable are appropriate. The time series of *GHI*, *Ws*, *Wd*, and *Ppv* exhibit standard deviation values exceeding the mean, a characteristic that can be attributed to the pronounced intermittency and randomness of these variables. In the case of *Ta*, *Tp*, and *Rh* demonstrate a more stable behavior with standard deviations lower than the mean. Additionally, it is important to acknowledge the presence of scale variations within the dataset, a factor that should be considered in subsequent analyses.

**Figure 3:**
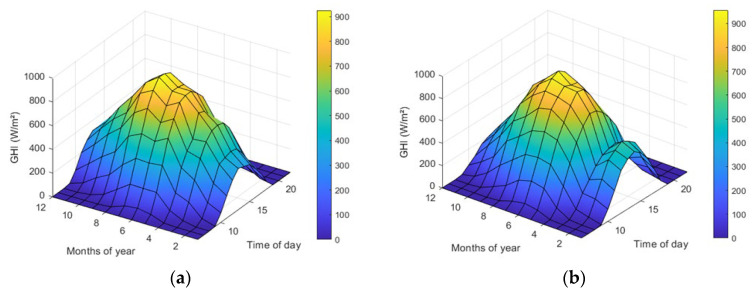
Average behavior of the *GHI* variable: (**a**) year 2021; and (**b**) year 2022.

It is also important to perform a graphical analysis of the data to identify trends and patterns. In this context, the *GHI* variable is used as a reference and analyze its average behavior concerning both the time of day and the month of the year, as illustrated in Figure 3.

Throughout the two years under examination, it is evident that the *GHI* levels are higher during the spring and summer seasons compared with the autumn and winter seasons. Additionally, variations in the average behavior of the same month across different years are apparent. This underscores the significance of having an extensive dataset to encompass a wider range of behavioral patterns for training the prediction model.

#### 2.2.2. Data Cleaning

Sometimes, the data may have several problems related to incorrect measurements, failures, and interruptions in the measurement equipment. This causes the available data to be corrupted and its quality to be affected, which can have a negative impact on model training [29]. To cope with these problems, a data cleaning algorithm, Algorithm 1, is applied to solve the problems related to outliers and missing data in each time series.
**Algorithm 1** Data Cleaning1:*Obtain each of the time series that make up the database*2:*Detect and replace outliers in each time series using step 3*3:*   time_serie = **filloutliers** (time_serie, fillmethod, findmethod) → (a method is applied that considers values that are more than three times the standard deviation of the mean as outliers and replaces them with the non-outlier closest to their position in the time series)*4:*Detect and replace missing values in each time series using step 5–6*5:*   time_serie = **fillmissing** (time_serie, movmethod, window) → (the interpolation method is applied based on the moving average with a sliding window of variable length. The size of the window is adjusted according to the amount of missing data in the time series)*6:*   time_serie = **fillmissing** (time_serie, method) → (In cases of larger missing data, the above technique can be combined with a piecewise cubic spline interpolation to achieve a smoother interpolation of the time series)*

**Figure 4:**
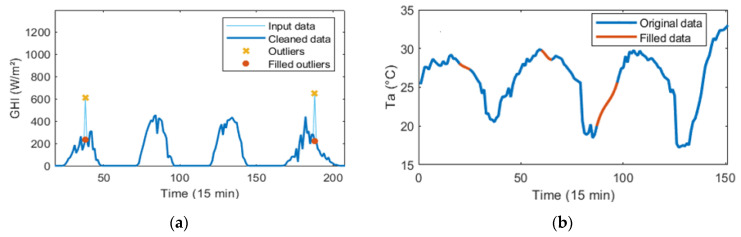
Results obtained by applying the data cleaning algorithm: (**a**) detection and correction of outliers in the *GHI* time series; and (**b**) correction of missing values in the *Ta* time series.

Figure 4 shows some results obtained by applying the data cleaning algorithm to the *GHI* time series (outlier’s detection and correction) and the *Ta* time series (missing value correction). The graphical results showed that the algorithm performs adequately in both cases.

#### 2.2.3. Elimination of the Night Values and Adjustment of the Time Resolution of the Data

PV energy production is zero during the night hours, making it possible to exclude these values without compromising the performance of the prediction model and the quality of its predictions. This is beneficial for model training, allowing the computational load and training time to be reduced. However, it should be noted that the sunrise and sunset times vary according to the seasons, and this is an important aspect that should be considered in order not to affect the quality of the dataset [30]. In this work, a filtering of the night values is implemented, taking into account these aspects, in order to guarantee the integrity of the data throughout the entire analyzed period.

At times, adjusting the temporal resolution of the available data is necessary to achieve optimal performance of the prediction model [29]. The data used in this work have a temporal resolution of 15 min, and the objective is to perform the PV power prediction of the following hour. To determine the most appropriate temporal resolution, a sensitivity analysis of the predictive model is performed. For this purpose, its performance is evaluated with different input data resolutions (15 min, 30 min, and 1 h) and the errors committed in each case are quantified. The most favorable results are obtained when using a temporal resolution of 30 min to forecast the *Ppv* of the following hour.

#### 2.2.4. Selection of Input Features to the Prediction Model

When working with a dataset that includes measurements of various variables, it is important to analyze and determine which variables have the most significant impact on PV power prediction. Adding more input features does not necessarily lead to better results. In fact, including inappropriate input features can introduce noise and reduce the quality of predictions. One commonly used method in the literature for selecting input features in a prediction model is Pearson’s correlation [31]. This method is based on determining the strength and direction of the linear relationship that exists between each independent variable and the target variable (represented in this case by the *Ppv*). The Pearson correlation coefficient (*PCC*) is calculated as follows:

(1) $$PCC=\frac{\frac{1}{n}\sum_{i=1}^{n}\left(x_{i}-\bar{x}\right)\left(y_{i}-\bar{y}\right)}{\sqrt{\frac{1}{n}\sum_{i=1}^{n}{\left(x_{i}-\bar{x}\right)}^{2}}\sqrt{\frac{1}{n}\sum_{i=1}^{n}{\left(y_{i}-\bar{y}\right)}^{2}}}$$

where $\bar{x}$ and $\bar{y}$ represent the mean of the vectors *x* and *y*, respectively; $x_{i}$ and $y_{i}$ indicate the value of measurement *i* for both vectors; and *n* represents the total number of measurements.

**Figure 5:**
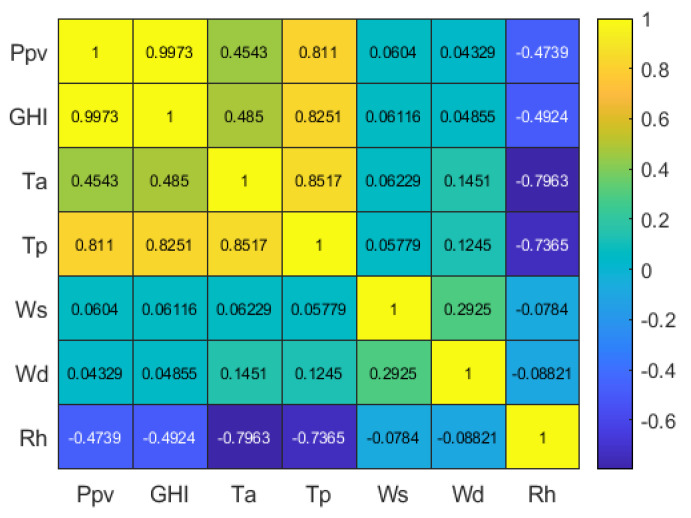
Pearson correlation matrix for the dataset analyzed.

Figure 5 displays the *PCC* matrix obtained derived from the analyzed database.

The results reveal a notably strong and positive correlation between the predictor variable (*GHI*) and the target variable (*Ppv*), confirming the great importance of this variable in PV energy forecasting. The variable *Tp* shows a strong and positive correlation, followed by the variable *Ta*, which shows a moderate and positive correlation. In the case of the variables *Ws* and *Wd*, their correlation levels are very low (practically zero) and it is not considered appropriate to use them as inputs in the forecasting model. Finally, the *Rh* variable has a moderate and negative correlation level with *Ppv*. This means that when one variable increases, the other decreases and vice versa. Given these findings, the variables *GHI*, *Tp*, *Ta*, *Rh*, and *Ppv* are selected as inputs to the predictive model.

#### 2.2.5. Selection of Lags in the Time Series and Adjustment of the Data Format

**Figure 6:**
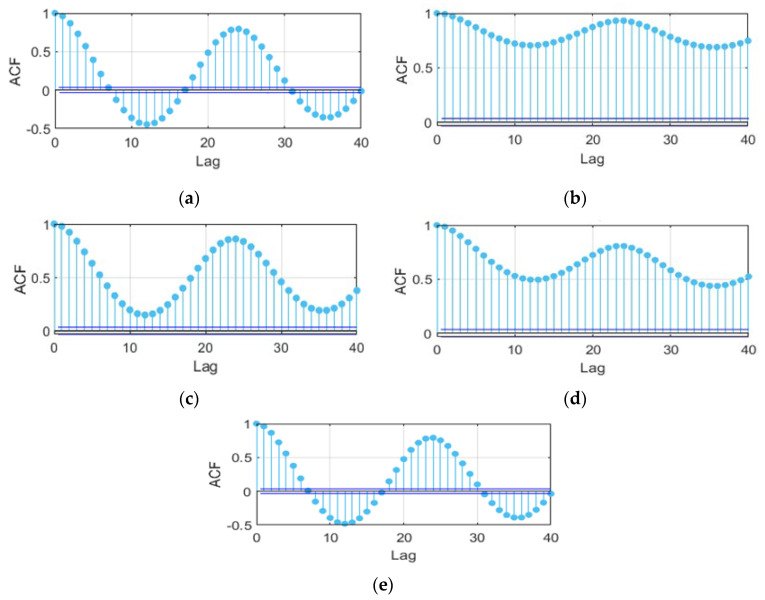
Autocorrelation function (ACF) for the input variables to the prediction model: (**a**) *GHI* time series; (**b**) *Ta* time series; (**c**) *Tp* time series; (**d**) *Rh* time series; and (**e**) *Ppv* time series.

The previous section outlines the process of selecting the most appropriate input characteristics for the presented PV forecasting problem. To incorporate a higher level of information in each input variable and improve the accuracy of the forecasts, an autocorrelation analysis is performed to know the existing relationship between a specific observation of the time series and its previous lags [14]. The results obtained for each selected input variable are shown in Figure 6. It is observed that the current value of each time series is strongly correlated with its previous lags. It is also evident that the time series under study have a seasonal behavior that repeats with a certain frequency, and the level of correlation decreases as the number of lags increases. To determine the appropriate number of lags to be used as inputs to the prediction model, a sensitivity analysis is performed again. The analysis starts with a small value (2 lags in this case), and progressively increases until reaching a larger value (40 lags). For each variant analyzed, the model is trained and the prediction errors made are determined. In this specific case, the best results are obtained by using the current value of each time series and five previous lags as inputs to the prediction model.

**Figure 7:**
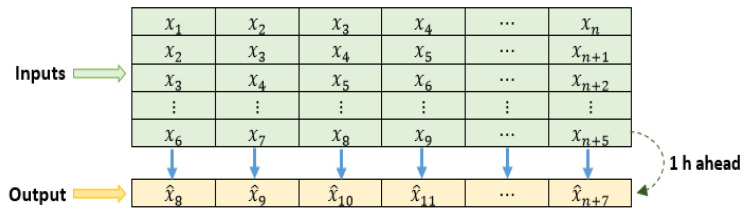
Structure used for the dataset.

Another important aspect to analyze is the adaptation of the data format. Figure 7 shows a general scheme of the structure used for the dataset. In this structure, the variable *x* represents the set of input characteristics, that is, $x=\left\{GHI,\;T_{a},T_{p},R_{h},P_{pv}\right\}$; $\hat{x}$ represents the forecast of the next hour’s *Ppv*. The variable *n* represents the number of observations used in the study, such that $n=N-\left(l+2\right)$, where *N* is the total number of observations in the dataset and *l* is the number of lags used. In this case, the data have been organized in such a way that there is a shift of two-time steps between the input variables and the corresponding output, with a time resolution of 30 min and a forecast horizon of one hour.

#### 2.2.6. Data Normalization

The dataset utilized in this work comprises several time series with varying value ranges. From the training perspective, it is advantageous to eliminate the differences in the scale of the data. This enhances the performance of the prediction model and its convergence speed [29]. For this purpose, the normalization method based on the minimum and maximum values is used, which allows scaling the data in the range [0, 1] and is expressed as follows:

(2) $$x_{norm}=\frac{x_{i}-x_{min}}{x_{max}-x_{min}}$$

where $x_{i}$ represents the original values of the variable; and $x_{min}$, $x_{max}$ represent the minimum and maximum values recorded.

At the end of the prediction phase, a reverse process must be applied to denormalize the predictions. In this way, comparisons can be made with actual *Ppv* measurements or with predictions from other prediction models.

#### 2.2.7. Data Splitting

**Figure 8:**
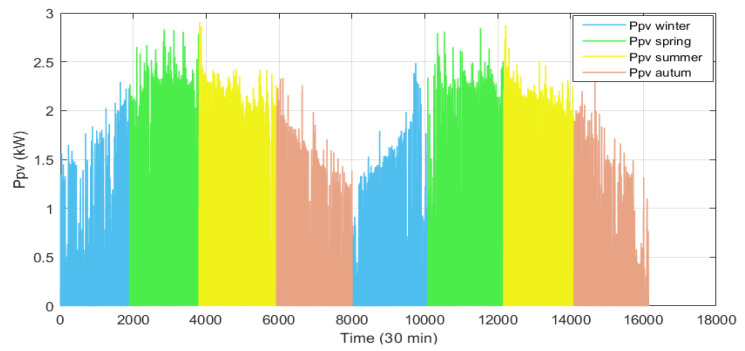
Division of the dataset according to the seasons of the year.

Data partitioning is also considered an important issue in PV forecasting. In this case, the available data are first divided according to the seasons of the year. Next, a dataset is created for each station, which may contain measurements from different years, as shown in Figure 8. Then, the data for each station are divided in such a way that 90% is used to train the model and the remaining 10% is used to test its performance and evaluate its learning ability.

### 2.3. Model Formulation and Hyperparameters Optimization by BOA

The third stage of the framework initially deals with aspects related to the formulation of the prediction model and its general structure. Subsequently, the hyperparameter optimization of the proposed model using BOA is described.

#### 2.3.1. Prediction Model Formulation

**Figure 9:**
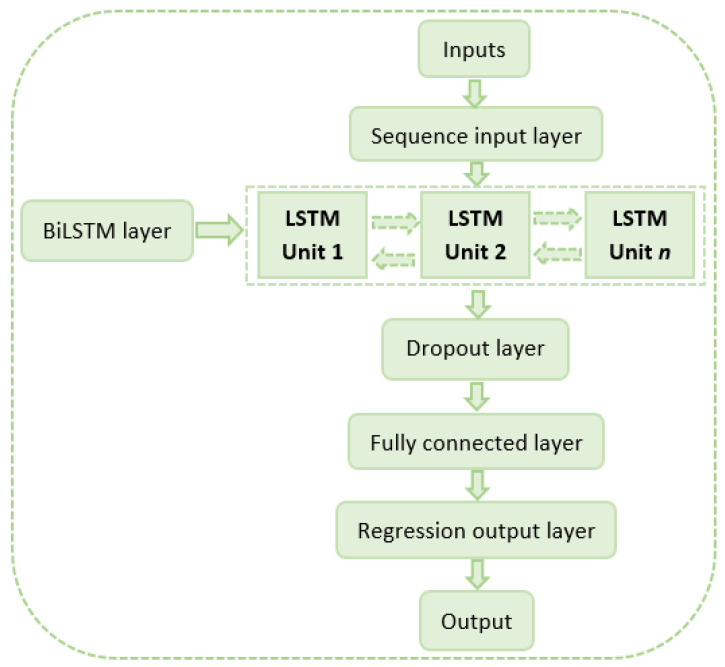
General architecture of the forecasting model.

The general architecture of the implemented prediction model is shown in Figure 9 and consists of the following elements: inputs, sequence input layer, BiLSTM layer, dropout layer, fully connected layer, regression output layer, and output.

In the preceding sections, the model inputs are defined, as well as the appropriate structure for the dataset, accounting for a specified number of lags in each time series. The sequence input layer is responsible for preparing the data to feed the BiLSTM layer, and is adjusted according to the number of inputs previously defined. The dropout layer is used to avoid overfitting problems; its operation is based on performing a random pruning of neurons while training the model. The use of this layer makes the resulting model more robust and adaptive. Then, the fully connected layer performs some linear and nonlinear transformations, and it is responsible for flattening the output. The adaptation is aligned with the number of model outputs. The regression output layer generates the final numerical prediction of the model, and the loss function calculation is performed in this layer. Finally, the output of the model is represented by the prediction of the *Ppv* of the next hour [32].

The BiLSTM layer consists of a number of LSTM units with specific connection patterns, so that the model can learn temporal relationships and behavioral patterns implicit in the input data. The LSTM model is described in detail in the literature [10,31]. In this case, the information processing during the learning process is performed in only one direction (forward). In the BiLSTM model, the data are processed in two directions (forward and backward). Thus, its performance and learning ability may be superior to other types of models in the same family [21,23]. The operation of the BiLSTM model can be represented as follows:

(3) $${\vec{h}}_{t}=LSTM\left(x_{t},\;{\vec{h}}_{t-1}\right)$$

(4) $${\overset{\leftarrow }{h}}_{t}=LSTM\left(x_{t},\;{\overset{\leftarrow }{h}}_{t+1}\right)$$

(5) $$y_{t}=W_{{\vec{h}}_{y}}{\vec{h}}_{t}+W_{{\overset{\leftarrow }{h}}_{y}}{\overset{\leftarrow }{h}}_{t}+b_{y}$$

where ${\vec{h}}_{t}$ and ${\overset{\leftarrow }{h}}_{t}$ represent the forward and backward hidden vectors, respectively; $x_{t}$ represents the model inputs at time *t*, while $y_{t}$ represents the corresponding output; $W_{{\vec{h}}_{y}}$ and $W_{{\overset{\leftarrow }{h}}_{y}}$ represent the LSTM model weights in both directions; and $b_{y}$ indicates the bias in the output layer.

#### 2.3.2. Hyperparameter Optimization Using BOA

**Figure 10:**
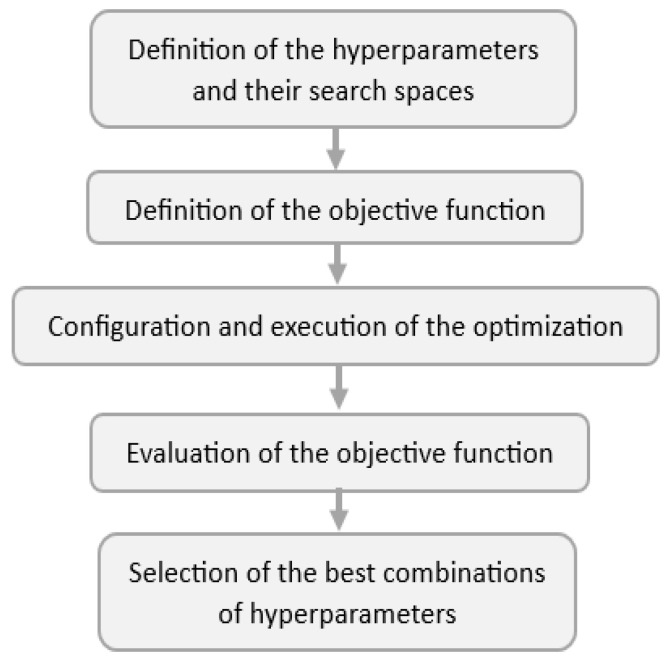
Main aspects considered in the BOA.

Bayesian optimization is suitable for problems of high dimensionality and with objective functions that are costly to evaluate, as is the case of hyperparameter optimization in DL models. BOA is based on Bayes’ theorem and uses two fundamental elements in its operation: the surrogate probability model and the acquisition function. The combination of both elements provides BOA an adaptive and probabilistic nature. In addition, it allows it to find efficient solutions in the search space with a smaller number of evaluations of the objective function and to adapt to limited computational resources [32,33]. An overview of the main aspects that integrate the BOA used in this work is presented in Figure 10.

The BOA has the mission to find the combination of hyperparameters that minimizes the objective function in a finite search space, in which a lower and upper bound is set for the variables [33]. The optimization problem can be formulated as:

(6) $$h^{*}=\underset{h\in H}{argmin}\;f\left(h\right)$$

where *H* corresponds to the search space defined for the hyperparameters involved in the optimization; *h* represents the combination of hyperparameters in *H*;$\;f\left(h\right)$ represents the objective function of the problem (in this case the mean square error (MSE) of validation is used); and $h^{*}$ corresponds to the optimized combination of hyperparameters obtained at the end of the optimization.

To facilitate the optimization process, several critical hyperparameters of the BiLSTM model are considered. Table 2 provides a concise overview of the defined search ranges for each hyperparameter. Constraining these search spaces to recommended value ranges serves to prevent them from converging towards unsuitable values. This not only reduces computational time but also enhances the algorithm’s efficiency [32].

In general, Bayesian optimization is considered an effective method for hyperparameter fitting in ML and DL models. Its use can contribute to increase the robustness, adaptability and accuracy of PV energy predictions. For a more detailed explanation of BOA, interested readers are referred to references [32,33,34].

## 3. Deterministic Predictions

This section deals with aspects related to the hyperparameter fit of the proposed model and the reference models, as well as the metrics used to evaluate the accuracy of the predictions made. The results obtained in the deterministic prediction of the hour-ahead PV power are also analyzed and discussed.

### 3.1. Fittings Obtained for the Proposed Model and the Reference Models

The previous section analyzed the aspects related to the formulation of the BiLSTM model and the optimization of its main hyperparameters using BOA. In this section, the fit values obtained for each hyperparameter considered in the optimization process are presented in a summarized and organized manner. In order to perform a more rigorous evaluation of the performance of the proposed model, it is considered appropriate to make comparisons with other types of models used in this field of research. For this purpose, an MLP-type ANN and an RF-type ensemble model are selected. Some hyperparameters of these models are also adjusted by BOA to ensure a reasonable balance in the comparisons. Since the data are divided into four groups corresponding to the seasons of the year, it is necessary to fit a specific prediction model for each group. Table 3 shows the values obtained for the hyperparameters of each model according to the seasons of the year analyzed.

It is also necessary to point out that for the training of the BiLSTM model, the adaptive moment estimation algorithm (Adam) is used and the training is carried out for 200 epochs. For the MLP model, the Levenberg–Marquardt (L-M) algorithm is used and the maximum number of iterations is set to 1000.

### 3.2. Metrics Used to Assess the Accuracy of Deterministic Predictions

A common practice when working with ML or DL models is to train the model with most of the available data and reserve a smaller portion to evaluate its performance on a new dataset. This evaluation of the model is performed through a set of metrics of different nature. In this work, the following metrics are used to evaluate the deterministic forecasts made by each model: mean absolute error (MAE) [35], normalized mean absolute error (nMAE) [35], root mean square error (RMSE) [35], and normalized root mean square error (nRMSE) [35]. The coefficient of determination (R^2^) [36] is also used to find out the level of fit between model predictions and actual PV power measurements. The purpose of using normalized metrics is to establish the necessary comparisons with other works in the literature that have used different databases. In these cases, it is not advisable to establish comparisons based on scale-dependent metrics such as MAE and RMSE.

### 3.3. Results Obtained in the Deterministic Predictions

**Figure 11:**
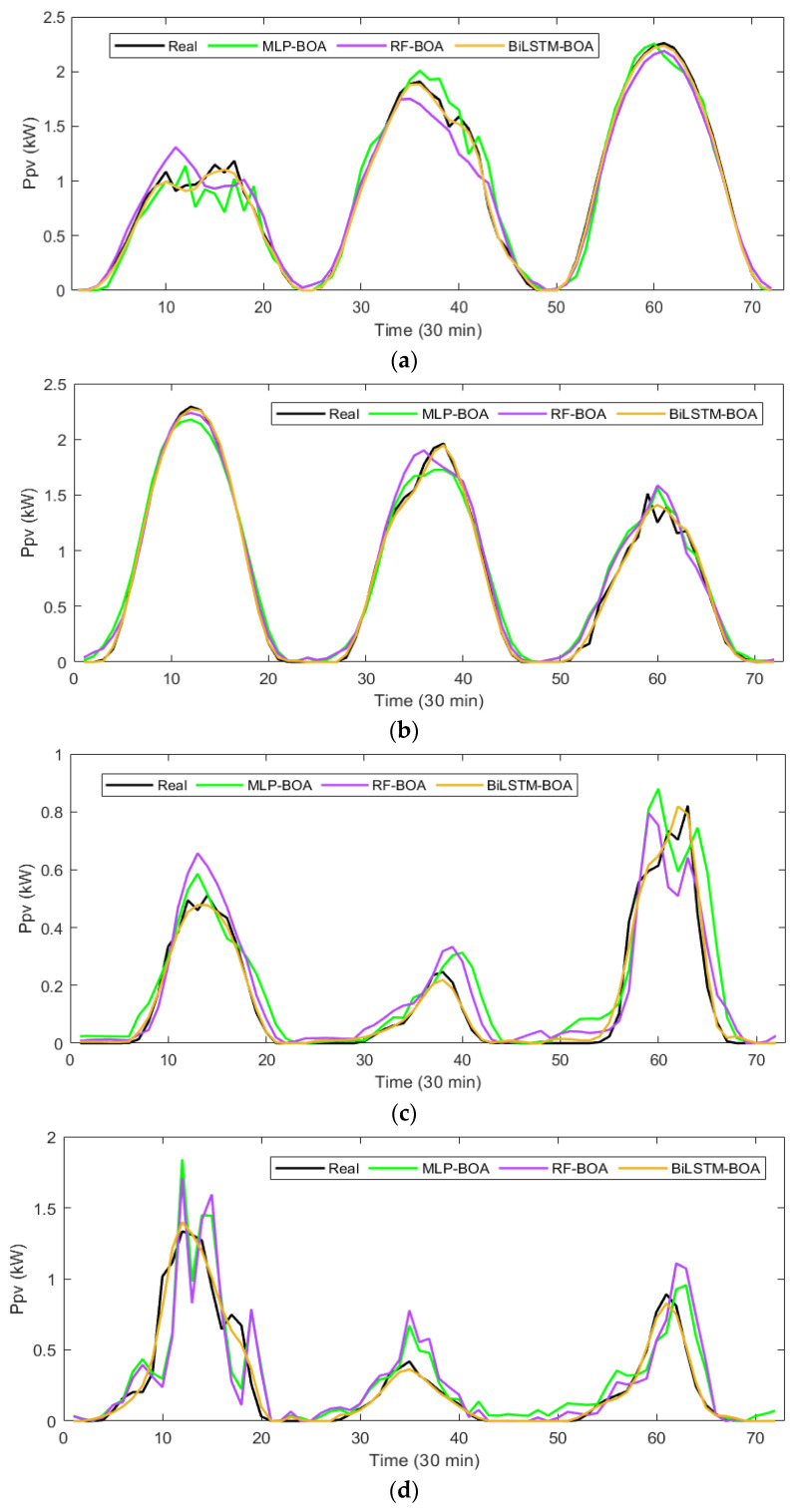
Comparison of the PV power deterministic predictions made by each model in different climatic conditions and seasons of the year: (**a**) spring season; (**b**) summer season; (**c**) autumn season; and (**d**) winter season.

To evaluate the predictive ability of each model in the deterministic prediction of the hour-ahead *Ppv*, its performance on the test dataset obtained for each season of the year is analyzed. These datasets represent a wide variety of behavioral patterns and weather conditions (sunny, partly sunny, cloudy, and rainy days, among others). Employing this predictive approach proves advantageous as it provides valuable insights into how each model behaves under various operational conditions. This analysis facilitates the determination of their effectiveness across different scenarios. Figure 11 displays the point predictions generated by the MLP-BOA, RF-BOA, and BiLSTM-BOA models, as well as the actual *Ppv* values recorded during the corresponding time period. In each season of the year, three days of different climatic behavior are randomly selected to assess the models’ performance in this type of prediction.

It is observed that all models show a better performance on days with more stable weather conditions (sunny and partly sunny days) and the opposite occurs on days where conditions are more dynamic and fluctuating (predominantly cloudy days).

Comparatively, the proposed BiLSTM-BOA model outperforms the reference models, consistently delivering predictions that closely align with the actual *Ppv* values recorded in each season of the year. These graphical results underscore the adaptability and flexibility of the BiLSTM-BOA model in accurately forecasting PV energy one hour in advance.

**Figure 12:**
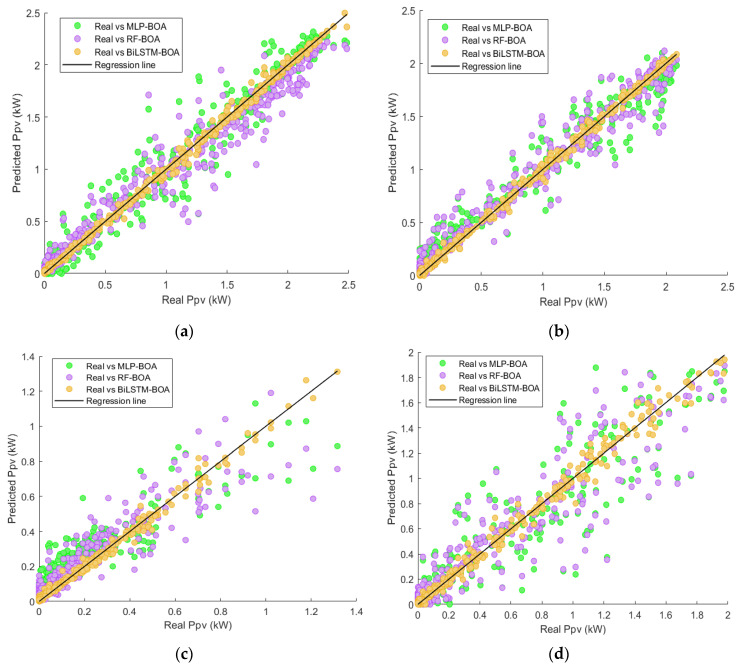
Scatter plots between the predictions of each model and the actual *Ppv* values in each season of the year: (**a**) spring season; (**b**) summer season; (**c**) autumn season; and (**d**) winter season.

Utilizing scatter plots is recommended for a visual examination of the relationships between model predictions and actual *Ppv* measurements. Figure 12 illustrates the results obtained for the test set across different seasons. The analysis discloses that during the summer, the models exhibit a more accurate alignment between predictions and measured values. In contrast, the spring season witnesses an augmented dispersion of observations compared with the preceding scenario. Likewise, in the autumn season, a decline in the predictive accuracy of MLP-BOA and RF-BOA models is discernible. Notably, the winter season manifests the highest level of dispersion, signifying increased uncertainties in predictions during this period.

In conclusion, the presented model consistently demonstrates robust performance across all examined scenarios, with predictions closely matching actual values. Even in the autumn and winter seasons, marked by dynamic solar energy behavior and heightened uncertainty, the model upholds a high level of prediction accuracy. This underscores the model’s effectiveness and resilience in addressing the intricacies of the forecasting task.

To quantify the errors made by each model in the deterministic prediction, several metrics recommended in the literature are utilized, namely: MAE, nMAE, RMSE, nRMSE and R^2^. The values obtained for each model and each season are summarized in Table 4. The interpretation of these metrics suggests that a model’s performance is better when it exhibits reduced values for (MAE, nMAE, RMSE, nRMSE) and high values of R^2^. From the analysis of the presented numerical results, it can be concluded that the BiLSTM model combined with hyperparameter optimization using BOA shows the best performance on each dataset. Taking, for example, the average values of the nMAE metric as a reference, the proposed model achieves an absolute error reduction of 3.55% with respect to the MLP-BOA model and 3.96% compared with the RF-BOA model; representing a decrease in prediction error of 75.03% and 77.01%, respectively. For the nRMSE metric, the trend is similar, with an absolute error reduction of 5.53% compared with the MLP-BOA model and 6.04% compared with the RF-BOA model; indicating an error reduction of 73.52% and 75.22% respectively. Finally, considering the average values of the R^2^ metric, it is observed that the proposed model shows an increase of 5.87% with respect to the MLP-BOA model and 6.17% with respect to the RF-BOA model.

**Figure 13:**
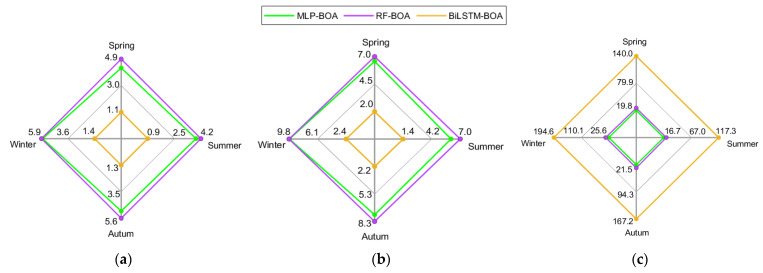
Comparison of the performance of MLP-BOA, RF-BOA, and BiLSTM-BOA models: (**a**) considering the nMAE; (**b**) considering the nRMSE; and (**c**) considering the training time.

Figure 13 graphically depicts the behavior of the nMAE and nRMSE metrics in each season of the year and for each of the models included in the study. The training time of the models in each available dataset is also plotted.

Regarding the normalized errors, the superiority of the proposed model in each test condition is evident. As for the training times, the MLP-BOA model shows the shortest times, closely followed by the RF-BOA model and finally by the BiLSTM-BOA model, which shows the highest times. In the latter case, since it is a DL model with a more complex architecture, the computational cost is higher. Nevertheless, the times obtained are considered reasonable, even for on-line forecasting applications that require frequent retraining of the model. On the other hand, the differences in training time for the different seasons may be related to variations in the hyperparameters of each model, since the proposed fitting is different for each dataset.

The final step in the deterministic forecasting stage is to compare the results obtained by the BiLSTM-BOA model with other work in the literature. In this case, several papers are selected that analyze similar prediction horizons and also use some normalized metrics to evaluate the quality of their predictions. However, it should be noted that each study uses a different dataset, which may have a relative influence on the performance of the predictive models. Table 5 shows the results obtained in each study.

Compared with the hybrid forecasting model (IF-ELM) presented in reference [12], the BiLSTM-BOA model presented in this work shows a slightly lower value in terms of R^2^, but achieves lower values of nMAE and nRMSE. However, reference [12] only uses as inputs to the model the time series of *Ppv* and its lags, while in the present study other exogenous variables are considered as inputs to the model. On the other hand, the data used in [12] come from an arid climate region, with more stable climatic conditions.

Compared with the models presented in reference [15], the prediction approach proposed in this work shows lower values of nMAE and nRMSE. Both studies use models belonging to the same family, although the characteristics of the data used and the model fitting strategies are different. With respect to the hybrid model (NARX-LSTM) presented in [17], the BiLSTM-BOA model shows very similar indicators in terms of nMAE and nRMSE, and slightly higher R^2^ values. Both models use as inputs a combination of exogenous variables and lags of *Ppv*, and in both cases optimization algorithms are used for hyperparameter fitting.

In the case of the hybrid model (SVR-CSO) of reference [35], the reported prediction errors are larger compared with the model proposed in this work. Finally, the hybrid model (GWO-GRNN) of reference [37] shows a lower nMAE value than that obtained by the BiLSTM-BOA model, although the R^2^ value of the latter is much higher. It should be noted that reference [37] normalizes its errors with respect to the nominal capacity of the plant, while in this work the normalization is performed with respect to the maximum value recorded in each test set. However, the normalization with respect to the nominal capacity of the plant is not recommended when the actual *Ppv* values are far from the nominal value since an underestimation of the prediction errors can be obtained.

From the previous comparisons, it can be concluded that the proposed model has a reasonable performance in predicting *Ppv* one hour in advance. In general, this model shows stable results, which are in line with other works in the literature that have used different state-of-the-art forecasting approaches to address this problem.

## 4. Interval Predictions

The utilization of prediction intervals is regarded as a suitable approach to effectively capture the uncertainties inherent in the deterministic predictions of the proposed model. In power systems characterized by a substantial penetration of photovoltaic (PV) power, these uncertainties in deterministic predictions can significantly impact the planning and operation of the system. Interval forecasts offer a viable solution to these challenges, enabling the consideration of a range of values within which the prediction is expected to fall, accompanied by a predefined nominal confidence level.

This section introduces the proposed algorithm designed for generating interval predictions and delineates the metrics employed to evaluate their accuracy. Following this, an analysis and discussion of the results obtained from this type of prediction are presented.

### 4.1. Proposed Algorithm for Obtaining Prediction Intervals

The proposed approach for obtaining the prediction intervals is based on combining the deterministic predictions of the BiLSTM-BOA model with a method known as bootstrap resampling [9,38], to obtain the lowest and upper prediction intervals for different confidence levels. The pseudocode of the proposed algorithm, Algorithm 2, is presented below.
**Algorithm 2** Obtaining prediction intervals1:*Obtain deterministic predictions from the proposed prediction model*2:*Determine the errors in the deterministic prediction*3:*Define the number of bootstrap samples to be generate (e.g., B = 10,000 → by using a larger number of bootstrap samples, a more diverse and representative bootstrap prediction matrix of the data sample can be obtained. However, using a very large value of this parameter can result in a considerable increase in algorithm execution time)*4:*Create a matrix of zeros of size n x B to store the bootstrap predictions (n represents the number of deterministic predictions)*5:*Follow steps (5–9) to obtain bootstrap predictions*6:***for** i = 1: B **do***7:   *Generate random samples of prediction errors with replacement to obtain bootstrap prediction errors*
8:   *Sum the bootstrap prediction errors obtained in the previous step with the deterministic predictions obtained in step 1, and store the results in the bootstrap prediction matrix defined in step 4*
9:***end***10:*Define the desired confidence percentile (e.g., 90% → this parameter is defined according to the user’s requirements)*11:*Determine the upper and lower prediction intervals based on the desired confidence percentile and the bootstrap predictions obtained in the previous step.*12:*Plot the actual values, the model’s deterministic predictions and the prediction intervals calculated in the previous step*

By calculating the PV power prediction intervals with a given confidence level, additional useful information is obtained about the possible range of values of this variable for a future time horizon. In this way, it is possible to improve the energy management of the system and avoid the occurrence of energy shortage or surplus problems, ensuring a more stable and safe operation of the PV system.

### 4.2. Metrics Used to Assess the Accuracy of Interval Predictions

For the evaluation of interval forecasts, two metrics recommended in reference [17,36] are selected, specifically the prediction interval coverage probability (PICP) [36] and the average coverage error (ACE) [17]. In this case the metrics are determined as follows:

(7) $$PICP=\frac{1}{T}\overset{T}{\underset{t=1}{\sum }}\epsilon_{t},\;where\;\epsilon_{t}=\left\{\begin{array}{c} 1\;if\;y_{t}\in \left[L_{t},\;U_{t}\right]\\ 0\;if\;y_{t}\notin \left[L_{t},\;U_{t}\right] \end{array}\right.$$

(8) ACE=PICP−PINC

where $U_{t}$ and $L_{t}$ correspond to the upper and lower bounds of the prediction interval respectively, $\epsilon_{t}$ represents a Boolean variable, and PINC represents the prediction interval nominal confidence.

### 4.3. Results Obtained in the Interval Predictions 

In this study, the methodology employed to establish the intervals is based on the bootstrap resampling method, as detailed in Section 4.1. While Section 3.3, generally demonstrates the effectiveness of the proposed model in various climatic and seasonal conditions, it is crucial to highlight that deterministic predictions, in many cases, deviate from the actual recorded values. This further underscores the relevance of adopting the interval approach, supported by the satisfactory results of the deterministic model, for proper planning of PV generation management.

**Figure 14:**
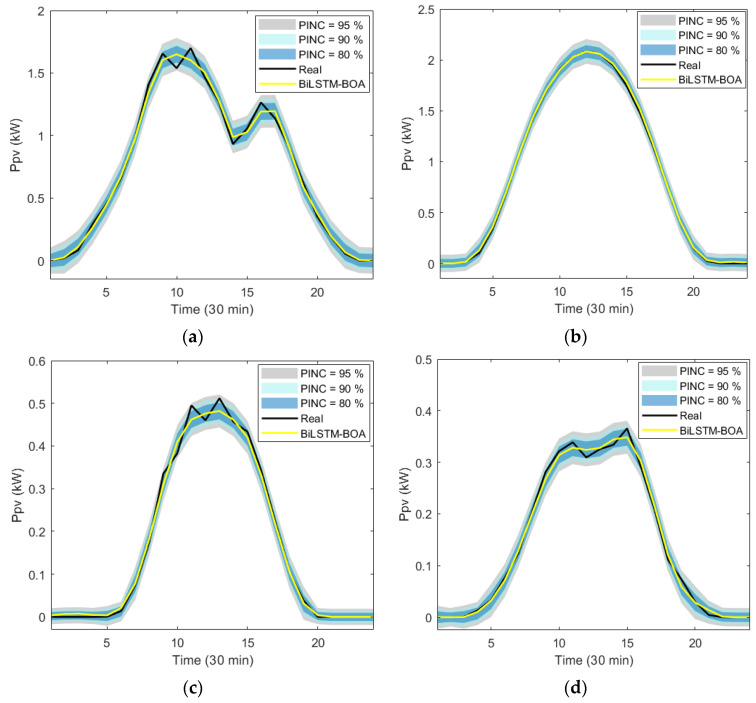
Prediction intervals obtained for a representative day of each season of the year, using different PINC values (80%, 90%, and 95%): (**a**) spring season; (**b**) summer season; (**c**) autumn season; and (**d**) winter season.

Figure 14 graphically represents the *Ppv* measurements, the deterministic predictions of the proposed model and the prediction intervals obtained from different PINC values (80%, 90% and 95%). In this case, the analysis is also carried out on the test dataset for each season of the year, and a representative day of each season is selected to demonstrate the effectiveness of the proposed strategy. In general, it is observed that the prediction intervals obtained in each case study adequately represent the defined PINCs. It can be seen that as the PINC is reduced, the width of the interval obtained is also reduced. The most favorable conditions occur on sunny days in the summer season where there is less uncertainty in the prediction. However, on the days selected for the remaining seasons, where a higher level of uncertainty is evident in the point predictions due to the effect of cloudiness, the calculated prediction intervals also adjust adequately to each operating condition analyzed.

To evaluate the quality of interval predictions, two metrics recommended for this type of prediction are used, namely the PICP and the ACE. In this case, it is recommended that the PICP values are as close as possible to the defined PINC, indicating that the calculated intervals do not overestimate or underestimate the defined confidence level. In the case of ACE, it is recommended that its values are close to zero. Table 6 shows the results obtained in predicting the intervals for each season of the year and for each PINC considered.

The PICP values obtained in each season, as well as the average values of this metric, are satisfactory and appropriately align with the defined confidence levels, which is beneficial from the perspective of system energy management. Concerning ACE values, it is observed that, in stations with more dynamic climatic conditions, these errors are higher compared with other stations with more stable climatic conditions. However, overall, the average ACE values are very close to zero, indicating a good adjustment of the prediction intervals in each case.

The most favorable results are obtained in the summer season with ACE values of 0.0219%, 0.0321%, and 0.0754%, corresponding to each analyzed confidence level (80%, 90%, and 95%) respectively. In the spring season, ACE values are 0.0614%, 0.0815%, and 0.1062%, representing an error increase of 64.34%, 60.61%, and 29.01%, respectively, compared with the previous case. For the autumn season, ACE values are 0.0924%, 0.1207%, and 0.1429%, equating to an error increase of 76.30%, 73.41%, and 47.24% compared with the summer season. Finally, the least favorable results are observed in the winter season, with ACE values of 0.1205%, 0.1454%, and 0.1831%, representing an error increase of 81.83%, 77.93%, and 58.82% compared with the baseline scenario.

This reaffirms the conclusions drawn from deterministic prediction, demonstrating that seasons with higher variability and intermittency in climatic conditions result in greater uncertainties in the BiLSTM-BOA model for PV energy forecasting.

## 5. Conclusions

This study introduces a BiLSTM-BOA model for forecasting one-hour-ahead PV power, leveraging historical data from a small PV installation at the Polytechnic School of the University of Alcala. Results from deterministic predictions highlight the positive impact of robust data preprocessing and optimization of fundamental model hyperparameters through BOA, enhancing predictive capability across diverse operational conditions. In comparative assessments with other reference models (MLP-BOA and RF-BOA), the proposed model consistently exhibits superior performance in tests conducted for each season and under various climatic conditions.

Examining the average performance of the nMAE metric, it is observed that the proposed model achieves error reductions in relative terms of 75.03% and 77.01% with respect to the MLP-BOA and RF-BOA models, respectively. A similar trend is observed in the nRMSE metric, with a relative error reduction of 73.52% with respect to MLP-BOA and 75.22% with respect to RF-BOA. Additionally, the results in terms of nMAE, nRMSE, and R^2^ align well with those reported in the literature for similar forecast horizons using advanced forecasting models.

The interval forecasts conducted through the bootstrap resampling method fit well with the established PINC levels and exhibit reduced ACE values in all analyzed cases. This results in a more flexible and robust final forecast, leveraging both methods and enabling more efficient management of the energy produced by PV systems. For this type of prediction, the most favorable results are obtained in the summer season, with an average ACE value of 0.0431% (including the three analyzed PINCs: 80%, 90%, and 95%). Using the summer season as a reference scenario, an average increase in ACE of 51.32% is observed for the spring season, while the autumn and winter seasons show average increases in ACE of 65.64% and 72.86%, respectively.

In summary, a comprehensive analysis of the obtained results suggests that the proposed forecasting strategy is effective and feasible for predicting the one hour-ahead PV power under various operational conditions, given the availability of necessary data. 

### Future Work

Future work will focus on applying the proposed strategy to other PV systems to obtain regional-scale predictions, as well as evaluating the impact of these forecasts on the operation of a power system with high penetration of PV energy. Furthermore, there is consideration for extending the development of the predictor to different time horizons, assessing the inclusion of meteorological variables as inputs, such as the forecasted cloud cover for the upcoming hours.

## Figures and Tables

**Table 1:** Statistical analysis of the available data.

Available Variables	Unit of Measure	Mean	Std.	Min. Value	Max. Value
Global horizontal irradiance (*GHI*)	[W/m^2^]	266.45	296.65	0	1144
Wind speed (*Ws*)	[m/s]	0.14	0.75	0	14.88
Wind direction (*Wd*)	[°]	35.38	73.65	0	266
Relative humidity (*Rh*)	[%]	47.02	18.75	6.01	86.75
Ambient temperature (*Ta*)	[°C]	19.49	9.49	−7.65	43.17
Panel temperature (*Tp*)	[°C]	22.96	14.54	−10.02	61.03
PV power (*Ppv*)	[kW]	0.68	0.72	0	2.87

**Table 2:** Hyperparameters optimization and their corresponding search spaces.

Hyperparameter	Search Space
Initial learning rate	[0.001 to 0.01]
Dropout value	[0.1 to 1]
Regularization factor L₂	[$10^{-5}$ to $10^{-2}$]
Mini-batch size	[32 to 256]
Number of units in the hidden layer	[20 to 200]

**Table 3:** Hyperparameters fitted by BOA for each model and for different datasets.

Models	Hyperparameter to Adjust	Adjustment Obtained for Each Season of the Year			
		Winter	Spring	Summer	Autumn
BiLSTM-BOA	Initial learning rate	0.005	0.004	0.004	0.005
	Dropout value	0.24	0.12	0.14	0.16
	Regularization factor L_2_	$3.7\times 10^{-4}$	$6.1\times 10^{-4}$	$1.4\times 10^{-4}$	$2.8\times 10^{-4}$
	Mini-batch size	32	32	64	32
	Units in the hidden layer	120	86	72	98
RF-BOA	Number of trees	484	495	468	498
	Number of predictors to sample	15	12	16	9
	Minimum leaf size	4	3	2	6
MLP-BOA	Activation function	logsig	logsig	logsig	logsig
	Target function	MSE	MSE	MSE	MSE
	Neurons in the hidden layer	58	49	38	53

**Table 4:** Errors made by each model in the deterministic prediction of *Ppv* in different seasons of the year.

Models	Metrics	Spring	Summer	Autum	Winter	Average
MLP-BOA	MAE (kW)	0.1050	0.0809	0.0663	0.1146	0.0917
	nMAE (%)	4.2280	3.8819	5.0380	5.7982	4.7365
	RMSE (kW)	0.1642	0.1272	0.0998	0.1935	0.1462
	nRMSE (%)	6.6095	6.0984	7.5847	9.7850	7.5194
	R^2^	0.9522	0.9703	0.9294	0.8861	0.9345
RF-BOA	MAE (kW)	0.1206	0.0874	0.0740	0.1165	0.0996
	nMAE (%)	4.8549	4.1893	5.6228	5.8990	5.1415
	RMSE (kW)	0.1749	0.1443	0.1096	0.1946	0.1559
	nRMSE (%)	7.0439	6.9221	8.3316	9.8408	8.0346
	R^2^	0.9487	0.9682	0.9246	0.8846	0.9315
BiLSTM-BOA	MAE (kW)	0.0278	0.0197	0.0169	0.0273	0.0229
	nMAE (%)	1.1193	0.9425	1.2848	1.3832	1.1825
	RMSE (kW)	0.0495	0.0286	0.0294	0.0467	0.0385
	nRMSE (%)	1.9945	1.3725	2.2349	2.3629	1.9912
	R^2^	0.9952	0.9965	0.9923	0.9889	0.9932

**Table 5:** Comparison of the results obtained in this research with other articles in the literature.

Reference and Year	Forecasting Method	Forecasting Horizon	Dataset Used and PV Plant Capacity	Inputs	Forecast Errors Obtained
[12], 2021	IF—ELM	1 h	DKASC, Alice Spring, Australia, site 20 *Pnom* = 5.04 kW	*Ppv* at time instant *t* and at 8 previous time instants	nMAE (%) = 1.6663 nRMSE (%) = 3.6082 R^2^ = 0.9954
[15], 2022	LSTM (vanilla, stacked, BiLSTM, autoencoder)	1 h	PV power plant in Bialystok, Poland *Pnom* = 317 kW	*GHI*, *Ppv*	MAE (kW) = 6.91 RMSE (kW) = 12.87 nMAE (%) = 2.18 nRMSE (%) = 4.06
[17], 2021	NARX—LSTM	1 h	DKASC, Alice Spring, Australia *Pnom* = 191.74 kW	*GHI*, *Ta*, *Rh*, *Wd*, diffuse horizontal radiation and lagged *Ppv*	nMAE (%) = 1.20 nRMSE (%) = 1.98 R^2^ = 0.9884
[35], 2023	SVR—Cuckoo search optimization (CSO)	1 h	PV power plant in Riyadh city, Saudi Arabia *Pnom* = 120 kW	*GHI*, *Ta*, direct normal irradiance (*DNI*), *Ppv* at the same hour on the previous day and hour of the day	MAE (kW) = 2.5661 RMSE (KW) = 4.4795 nMAE (%) = 2.4871 nRMSE (%) = 4.3417
[37], 2022	Grey wolf optimization (GWO)—based general regression neural network (GRNN)	1 h	PV power plant in Taiwan *Pnom* = 200 kW	*GHI*, *Ws*, *Ta*, *Rh*, rainfall, time and climatic groups obtained by means of a self-organizing map (SOM)	MAE (kW) = 1.884 RMSE (kW) = 4.052 nMAE (%) = 0.942 R^2^ = 0.955
This paper	BiLSTM—BOA	1 h	PV power plant in Polytechnic School of the University of Alcala, Spain *Pnom* = 2.97 kW	*GHI*, *Tp*, *Ta*, *Rh* and *Ppv*	MAE (kW) = 0.0229 RMSE (kW) = 0.0385 nMAE (%) = 1.1825 nRMSE (%) = 1.9912 R^2^ = 0.9932

**Table 6:** Evaluation of interval predictions in different seasons for different PINC values.

Season	PICP (%)			ACE (%)		
	PINC = 80%	PINC = 90%	PINC = 95%	PINC = 80%	PINC = 90%	PINC = 95%
Spring	80.0614	90.0815	95.1062	0.0614	0.0815	0.1062
Summer	80.0219	90.0321	95.0754	0.0219	0.0321	0.0754
Autum	80.0924	90.1207	95.1429	0.0924	0.1207	0.1429
Winter	80.1205	90.1454	95.1831	0.1205	0.1454	0.1831
Average	80.0741	90.0949	95.1269	0.0741	0.0949	0.1269

## Data Availability

The dataset used in this work is available upon request.
